# A personalized mRNA vaccine has exhibited potential in the treatment of pancreatic cancer

**DOI:** 10.1007/s44178-023-00042-z

**Published:** 2023-06-08

**Authors:** Ning Kang, Si Zhang, Yuzhuo Wang

**Affiliations:** 1 Department of Experimental Therapeutics, BC Cancer Agency, Vancouver, BC Canada; 2 Vancouver Prostate Centre, Vancouver, BC Canada; 3 Department of Urologic Sciences, University of British Columbia, Vancouver, BC Canada

**Keywords:** Pancreatic cancer, mRNA cancer vaccines, Lipid nanoparticles, Neoantigens, Clinical trials

## Abstract

This commentary discusses a ground-breaking study on the use of personalized mRNA cancer vaccines for treating pancreatic ductal adenocarcinoma (PDAC), a highly malignant form of cancer. The study, which capitalizes on lipid nanoparticles for mRNA vaccine delivery, aims to induce an immune response against patient-specific neoantigens and offers a potential ray of hope for improving patient prognosis. Initial results from a Phase 1 clinical trial indicated a significant T cell response in half of the subjects, opening new avenues for PDAC treatment. However, despite the promising nature of these findings, the commentary emphasizes the challenges that remain. These include the complexity of identifying suitable antigens, the possibility of tumor immune escape, and the requirement for extensive large-scale trials to confirm long-term safety and efficacy. This commentary underscores the transformative potential of mRNA technology in oncology while highlighting the hurdles that need to be overcome for its widespread adoption.

Pancreatic cancer, particularly pancreatic ductal adenocarcinoma (PDAC), which accounts for over 95% of all pancreatic cancer cases, is notoriously challenging to diagnose and treat due to its high malignancy. Its incidence has been notably rising in recent years, with the majority of cases only diagnosed at advanced stages, earning it the chilling moniker of "the king of all cancers" due to its low survival rates and poor prognosis [1].

As it stands, surgery is the only effective method of treating PDAC. Still, approximately 90% of patients experience relapse within 7–9 months post-operation (median time), with a 5-year overall survival rate standing at a mere 8–10%. Adjuvant chemotherapy after surgery can delay PDAC recurrence, but almost 80% of patients still relapse around 14 months post-operation, and the 5-year overall survival rate is less than 30%. Radiation, biological agents, and targeted therapies have shown no significant efficacy, and PDAC has proven almost entirely insensitive to immune checkpoint inhibitors with a response rate less than 5%. This insensitivity can be partially attributed to the low mutation rate of PDAC and the consequent scarcity of neoantigens, resulting in weak antigenicity of PDAC.

However, recent studies have indicated that most PDACs actually harbor more neoantigens than previously predicted. Moreover, research on long-term PDAC survivors suggests that neoantigens can potentially stimulate T cells within PDAC. Therefore, strategies to deliver these neoantigens might induce neoantigen-specific T cells, thereby improving patient prognosis [2].

mRNA cancer vaccines represent an exciting frontier in oncology, harnessing the power of the immune system to recognize and destroy cancer cells. The approach builds on the success of mRNA technology used in COVID-19 vaccines, such as those developed by Pfizer-BioNTech [3] and Moderna [4]. But, the application in cancer treatment comes with its unique set of challenges and opportunities.

The fundamental idea behind mRNA cancer vaccines is the ability to direct the immune system towards specific tumor-associated antigens (TAAs) or neoantigens [5]. TAAs are proteins expressed at high levels in cancer cells, while neoantigens are entirely new proteins resulting from the mutations in cancer cells. By using mRNA to encode these specific antigens, the vaccines aim to stimulate an immune response that specifically targets and kills cancer cells, while leaving healthy cells unharmed.

One major advantage of mRNA vaccines is their flexibility. The mRNA sequence can be quickly and easily altered in response to the unique antigenic profile of a patient's tumor, allowing for a highly personalized treatment approach. This is particularly relevant in the context of neoantigens, which can vary widely between individuals and even within different regions of the same tumor.

Lipid nanoparticles (LNPs) have emerged as a critical component in the development and success of mRNA vaccines, most notably demonstrated in the COVID-19 vaccines developed by Pfizer-BioNTech and Moderna. They play a central role in the effective delivery of the mRNA payload to cells, promoting the production of the protein that will ultimately trigger an immune response.

mRNA is inherently unstable and prone to degradation. Furthermore, it's negatively charged, which prevents it from easily crossing the lipid bilayer of cells. LNPs help to overcome these challenges by encapsulating the mRNA, protecting it from premature degradation, and facilitating its delivery into the cytoplasm of cells [6].

A key component for mRNA LNP is an ionizable cationic lipid, which carries a positive charge at low pH (as in the conditions during LNP synthesis) but is neutrally charged at physiological pH. This property allows the LNPs to form complexes with the negatively charged mRNA during the formulation process and to release the mRNA once inside the cell [6].

Other components of LNPs include phospholipids, cholesterol, and PEGylated lipids. The phospholipids and cholesterol contribute to the stability and fluidity of the nanoparticles, while the PEGylated lipids extend their circulation time in the body by reducing clearance by the immune system.

Moreover, the small size of LNPs (typically around 100 nm in diameter) allows them to be taken up by cells through endocytosis and captured in the endosome. The acidic environment of the endosome causes the ionizable lipid to take on a positive charge, which leads to localized disruption of the endosomal membrane and release of the mRNA into the cytoplasm, where it can be translated into protein [6].

The development and success of LNP technology has been a game changer for mRNA vaccines, enabling the successful delivery of mRNA into cells and contributing to the unprecedented speed of COVID-19 vaccine development. Future applications of this technology are expected to extend beyond vaccines to other areas of medicine, such as gene therapy and cancer immunotherapy [7].

Personalized RNA neoantigen vaccines: On May 10, 2023, a research team comprised of the Memorial Sloan Kettering Cancer Center, BioNTech, and Genentech (a subsidiary of Roche) published a paper in the prestigious academic journal Nature titled "Personalized RNA neoantigen vaccines stimulate T cells in pancreatic cancer" [2].

As aforementioned, PDAC is generally recognized as a weakly antigenic tumor with few infiltrating T cells [1]. However, long-term PDAC survivors have been observed to have spontaneous T cell responses to tumor-specific neoantigens, although these responses vary among patients. Therefore, the research team aims to develop a personalized mRNA vaccine that induces neoantigen-specific T cells to eliminate tumor metastasis and delay recurrence, thereby providing clinical benefits to PDAC patients after surgical resection.

Therapeutic mRNA vaccine technology has paved the way for the development of personalized neoantigen vaccines. Neoantigens can be identified by individual tumor exosome sequencing, and mRNA vaccines targeting multiple patient-specific neoantigens can be rapidly produced for personalized vaccination [8, 9]. Upon administration, mRNA can be taken up and translated by antigen-presenting cells (APCs). Expressed neoantigens can then be presented via major histocompatibility complex (MHC) molecules. This leads to the activation and expansion of neoantigen-specific CD4^+^ and CD8^+^ T cells, resulting in anti-tumor immune responses [10]. Importantly, mRNA vaccines can be delivered using LNPs, which have been extensively validated clinically [7].

In this Phase 1 clinical trial, the research team injected a personalized mRNA vaccine—autogene cevumeran—expressing up to 20 neoantigens, delivered using LNPs via intravenous injection, in 16 PDAC patients after surgical resection, in combination with chemotherapy (mFOLFIRINOX regimen) and immune checkpoint therapy (anti-PD-L1 monoclonal antibody). A significant T cell response was observed in 50% of the patients, indicating that this personalized mRNA vaccine could trigger enhanced immune responses.

These results highlight the potential of personalized mRNA vaccines in treating PDAC, providing evidence of their general effectiveness as a therapeutic tool. Even with the low mutation rate of PDAC, mRNA vaccines can still induce T cell activity against new antigens produced by PDAC. The research team states that despite the limited sample size, these preliminary results suggest the need for broader clinical studies on this PDAC vaccine.

In summary, this study provides preliminary clinical trial evidence that this personalized mRNA neoantigen vaccine, Autogene cevumeran (BNT122), when used in conjunction with Atezolizumab (anti-PD-L1 monoclonal antibody) and mFOLFIRINOX (the standard adjuvant chemotherapy regimen after PDAC surgery), induces significant T cell activity in PDAC patients who have undergone surgical resection and are at risk of delayed recurrence.

This personalized mRNA neoantigen vaccine, Autogene cevumeran (BNT122) [9], jointly developed by BioNTech and Genentech, a subsidiary of Roche, is currently in Phase 2 clinical trials for the treatment of melanoma (NCT03815058) and colorectal cancer (NCT04486378). Clinical trials for solid tumors (NCT03289962), such as PDAC discussed in this paper (NCT04161755), are about to enter Phase 2.

Early clinical trials of mRNA cancer vaccines have shown promise [10]. However, several challenges remain. Firstly, the identification of suitable antigens is not straightforward and requires extensive genetic sequencing and bioinformatic analysis. Secondly, tumors often have mechanisms to evade the immune system, such as the expression of checkpoint molecules that inhibit T cell activity. This means that mRNA cancer vaccines are likely to be most effective when combined with other therapies, such as immune checkpoint inhibitors.

Finally, as with any new therapeutic approach, the long-term safety and efficacy of mRNA cancer vaccines remain to be established through large-scale clinical trials. Despite these challenges, the potential of mRNA technology to revolutionize cancer treatment is clear, and this is an area of intense research and development.

## Data Availability

All data from public domains.
